# Hydrogen sulfide protects retina from blue light-induced photodamage and degeneration via inhibiting ROS-mediated ER stress-CHOP apoptosis signal

**DOI:** 10.1080/13510002.2022.2069534

**Published:** 2022-04-28

**Authors:** Sen Zhu, Xuan Li, Bingrong Dang, Fen Wu, Kexin Gou, Chunming Wang, Changjun Lin

**Affiliations:** a School of Life Sciences, Lanzhou University, Lanzhou, People’s Republic of China; b Lanzhou University Second Hospital, Lanzhou, People’s Republic of China; c Chinese Academy of Sciences, Institute of Modern Physics, Lanzhou, People’s Republic of China

**Keywords:** H_2_S, photodamage, retinal degeneration, oxidative damage, endoplasmic reticulum stress, reactive oxygen species‌, apoptosis, ARPE-19 cells

## Abstract

**Background:** Hydrogen sulfide (H_2_S) is a small reducing gas molecule with various biological functions such as anti-oxidative, anti-apoptotic and anti-inflammatory activities. In this study, we investigated the therapeutic effects of exogenous H_2_S in the experimental models of retinal photodamage in vivo and in vitro.

**Methods:** Rats with open eyelids were pretreated with H_2_S (80~120 μmol/kg) for 10 days and then continuously exposed to blue light (435~445nm, 11.2W/m2) for 8 h to establish in vivo experimental model. ARPE-19 cells were pretreated with H_2_S and then exposed to blue light to establish in vitro experimental model.

**Results:** In vivo experiments, H_2_S significantly ameliorated blue light-induced retinal oxidative stress, apoptosis and degeneration. Moreover, H_2_S inhibited the activation of blue light-induced endoplasmic reticulum (ER) stress CHOP apoptotic signaling. In vitro experiments, H_2_S improved blue light-induced oxidative stress and oxidative damage. H_2_S inhibited ROS-mediated activation of ER stress CHOP apoptotic signaling. H_2_S alleviated blue light-induced apoptosis and increases cell viability. The ER stress inhibitor 4-PBA alleviated blue light-induced apoptosis and increases cell viability.

**Conclusion:** Taken together, these results indicate that H_2_S can inhibit ROS-mediated ER stress-CHOP apoptosis signal, thereby alleviating blue light-triggered retinal apoptosis and degeneration.

## Introduction

With the wide application of electronic products in recent years, more and more teenagers’ eyesight are affected, but people often attribute vision degradation to myopia [1]. However, the photochemical damage of the retina caused by blue light released from electronic screens is often overlooked, which is also another key factor to the vision loss in teenagers [2,3]. Blue light is visible light with wavelengths second only to ultraviolet light, and it penetrates into human tissues more strongly than ultraviolet light [4]. Blue light can significantly inhibit viability and induce cell death in ARPE-19 cells [5]. In addition, the retina is the most oxygen-consuming tissue and is often exposed to high oxidative stress [6]. Blue light can induce ARPE-19 cells to produce a large amount of reactive oxygen species (ROS) and cause intracellular oxidative stress and oxidative damage [7]. Blue light-induced ROS can activate apoptosis signals, such as ER stress-CHOP apoptosis signal, thereby inducing cell apoptosis in ARPE-19 cells [8]. Furthermore, *in vivo*, blue light-induced oxidative stress and apoptosis are crucial impact factors to blue light-induced retinal damage and degeneration [9].

Hydrogen sulfide (H_2_S) is a reducing gas molecule in cells and can be synthesized by a variety of enzymes [10]. H_2_S is recognized to alleviate the development of a variety of physiological and pathological diseases through antioxidant effects [11]. Moreover, small gas molecules such as H2S, nitric oxide (NO), and carbon monoxide (CO) have been shown to have great potentials in treating ophthalmic diseases by regulating vascular tone, which has been confirmed in fundus vascular tone [12,13]. Furthermore, in our previous research, we have found that H_2_S can alleviate intracellular oxidative stress and oxidative damage, restore cell viability, and reduce oxidative stress-mediated cell death and apoptosis[14]. These studies suggest that exogenous H_2_S may play an important role in protecting retinal damage and treating ophthalmic diseases. However, whether H_2_S can protect retina from blue light-induced oxidative stress and photodamage is still unclear, no matter *in vitro* or *in vivo* evidence.

In this study, we investigated the effects of exogenous H_2_S on blue light-induced retinal oxidative stress, ER stress and apoptosis *in vivo* and *in vivo*, and explored the feasibility of exogenous H_2_S as a drug for the prevention and treatment of retinal degeneration or retinal diseases.

## 2. Material and methods

### 2.1. Materials

ARPE-19 cell line was purchased from the China Center of Type Culture Collection (Shanghai, China). DMEM medium was obtained from Viva-Cell (Shanghai, China). Fetal bovine serum (FBS) was purchased from Si-Ji-Qing (Hangzhou, China). Sodium hydrosulfide (NaHS, 68%∼72%) was obtained from Macklin (Shanghai, China). LED blue light (435–445 nm, 11.2 W/cm^2^) device was purchased from JDL company (Hangzhou, China). 2′,7′-Dichlorofluorescin diacetate (DCFH-DA) was purchased from Sigma-Aldrich (St. Louis, MO, USA). Anti-GRP78, anti-CHOP and anti-β-Actin antibodies were purchased from Beyotime (Shanghai, China). Annexin V-FITC/PI apoptosis detection kit was purchased from Beyotime (Shanghai, China). Superoxide dismutase (SOD) and malondialdehyde (MDA) detection kits were purchased from Nanjing Jiancheng Bioengineering Institute (Nanjing, China). 3-(4,5-dimethylthiazol-3-yl)-2,5-diphenyl tetrazolium bromide (MTT) was purchased form Beyotime (Shanghai, China). TUNEL kit, Antigen Repair kit and FAS eyeball fixative were purchased from Servicebio (Wuhan, China). Other common reagents used in the study are of analytical purity grade.

### 2.2. Cell culture and treatment

ARPE-19 cells were cultured in DMEM high glucose medium supplemented with 10% FBS, 100 U/ml penicillin, and 100 mg/ml streptomycin at 37°C in air containing 5% CO_2_. When the cell confluence reached 85%–90%, the cells were digested with trypsin, collected by centrifugation, washed three times with PBS, resuspended in complete medium. Subsequently, the resuspended cells were then divided into two parts for further culture, or seeded into well plates for subsequent experiments. ARPE-19 cells were pretreated with or without NaHS (600/800 μM) for 1 h and then exposed to blue light (435∼445 nm, 11.2 W/m^2^) for 4 h, and the treated cells were subjected to subsequent experiments. NaHS was dissolved in DMEM medium to make a 100× stock solution, and then diluted and added into cell culture medium for incubation. Exogenous H_2_S (NaHS as donor) did not have obvious effect on the pH value of cell culture medium (Figure S1).

### 2.3. Analysis of cell viability

MTT assay was used to test the effects of blue light and NaHS on cell viability. ARPE-19 cells were pretreated with NaHS for 1 h and subsequently exposed to blue light for 4 h, and then cultured for another 24 h. After that, cell medium was replaced with equal complete medium containing 1 mg/mL MTT and cells were incubated at 37°C for another 4 h. Then, the medium was poured off, and DMSO was added to dissolve crystal violet. The absorbance was detected at 470 nm by the microplate reader (Varioskan Flash, Thermo).

### 2.4. Apoptosis analysis

ARPE-19 cells were pretreated with NaHS for 1 h and exposed to blue light for 4 h, and then cultured for another 24 h. Annexin V-FITC/PI apoptosis Kit was used to detect apoptosis in ARPE-19 cells, according to the manufacturer’s instructions. Analysis of apoptotic cells by flow cytometry (LSP Fortessa, BD) and inverted fluorescent microscopy (IX71, Olympus).

### 2.5. Western blot analysis

ARPE-19 cells were pretreated with NaHS for 1 h and exposed to blue light for 4 h, and subsequently cultured for another 24 h. Then, cells were collected and lysed in RIPA buffer containing 1% protease inhibitor PMSF for 30 min on ice. The subsequent procedures were consistent with those in our previous study [14].

### 2.6. Measurement of ROS level

ARPE-19 cells (3 × 10^5^) were seeded in 6-well plates for 24 h. The cells were pretreated with NaHS for 1 h and then exposed blue light for 2 h. Subsequently, the DCFH-DA fluorescent probe was added immediately and incubated with ARPE-19 cells in the dark for 20 min, and then flow cytometry was used to detect ROS fluorescence. In animal experiments, cryosections (5 μm thin) of eye tissues were labeled with DHE for intraretinal ROS and subsequently detected by fluorescent microscopy (IX71, Olympus). Three eyes per group were detected.

### 2.7. Analysis of MDA level and SOD activity

ARPE-19 cells (3 × 10^5^) were seeded in 6-well plates for 24 h. Then ARPE-19 cells were pretreated with NaHS for 1 h and exposed to blue light for 4 h, and subsequently cultured for another 24 h. MDA content and SOD activity were detected with the kits, according to the manufacturer’s instructions. In brief, MDA content was determined by its reaction with thiobarbituric acid (TBA) to form a colorimetric product, which could be detected at 532 nm wavelength. The intracellular SOD activity was detected at a wavelength of 450 nm.

### 2.8. Animal treatment

Thirty female Sprague–Dawley (SD) rats (six weeks old) were obtained from the Laboratory Animal Center of Lanzhou University, and the SD rats were randomly divided into five groups (six rats/group): Ctr, Control group; BL, Blue light irradiation group; BL + Ns, Blue light irradiation and intraperitoneal injection of normal saline group; BL + H_2_S (80 μmol/kg), Blue light irradiation and intraperitoneal injection of 80 μmol/kg NaHS group (NaHS dissolved in normal saline); BL+ H_2_S (120 μmol/kg), Blue light irradiation and intraperitoneal injection of 120 μmol/kg NaHS group. Except for the Control group and the Blue light irradiation group, rats in other groups were injected intraperitoneally with normal saline or NaHS for 10 consecutive days (once a day). Then, except for the Control group, rats in other groups were continuously exposed to blue light (LED, 435–445 nm, 11.2 W/cm^2^) for 8 h. During irradiation, the rats were fixed at an equidistant (35 cm) from the blue light device. All rats were undergone eyelid surgery on the 3rd day before irradiation to ensure that the irradiated retina received sufficient blue light irradiation dose. After that, the rats were euthanatized by intraperitoneal injection of sodium pentobarbital at 200 mg/kg and tissue samples were obtained for subsequent experiments. Each group of animals were housed in different stainless-steel cages, which were placed in air-conditioned in the laboratory (room temperature: 20°C–25°C; humidity: 45%–65%), in a 12-hour light–dark cycle. All rats were treated in accordance with the guidelines established by the Association for Research in Vision and Ophthalmology (ARVO) on animal use in ophthalmological and visual research, and all procedures were approved by the Ethics Committee of School of Life Sciences, Lanzhou University (EAF2021001).

### 2.9. Eyelid enlargement surgery

Before surgery, the rat was anesthetized by isoflurane. We observed its respiration depth, frequency, and body dynamic responses, in order to evaluate the anesthesia satisfaction. After the preparation was finished, the rat was placed in a lateral recumbent position, disinfected and draped. A longitudinal incision was made from the outer canthus perpendicular to the palpebral fissure, and extended laterally to the ear. To form a triangular incision, we needed to cut off part of the connective tissue of the upper and lower eyelids and the side of the eyeball. Then, we repeated this operation to enlarge the contralateral eyelid. This surgery allowed the rat to get more light into eyes. The whole process took about 10 min. After the operation, the rat was rewarmed. Vital signs were closely observed until the rat resuscitated and was put back to the incubator. The operation did not damage the ocular muscle tissue, lacrimal sac and lacrimal gland in rats, and according to our observations, the blink reflex was not affected after the recovery period.

### 2.10. Morphology analysis of the retina (Hematoxylin and Eosin staining, H&E)

After the rats were euthanized, the eyeballs were immediately removed and fixed with FAS eyeball fixative. Subsequently, the eyeballs were embedded in paraffin, and cut into 5 μm thin slices along the vertical meridian of eyeballs. Then, the retinal tissues were stained with hematoxylin and eosin and photographed under a fluorescent microscope (HBO100, Zeiss). The retinal thickness measurement was extended from the optic disc to both ends, and each 50 μm was photographed. Three images were obtained in each direction, and the average value was calculated as the representative value of thickness. The thickness of inner nuclear layer (INL), outer nuclear layer (ONL), retinal pigment epithelium layer (RPE) and entire retinal layer was analyzed by the ImageJ software. One eyeball was taken from each of six rats in each group for analysis. Furthermore, images of TUNEL assay, ROS assay and immunohistochemical assay were obtained by photographing near the optic disc.

### 2.11. TUNEL assay

Eyeball tissue was cryosectioned and cut into 5 μm thin slices. The frozen-slices were restored to room temperature and dried in air. Then the slices were fixed in cold acetone for 10 min and dried in air again. Subsequently, the slices were washed three times for 5 min each with PBS (pH 7.4) in a rocker device. Then, apoptotic cells in the retina were detected by the TUNEL kit, according to the manufacturer’s instruction. The apoptotic cells were visualized under a fluorescent microscope (Eclipse C1, Nikon). Images of retinal cell apoptosis were obtained by photographing near the optic disc. Three eyes per group were used for analysis.

### 2.12. Immunohistochemistry

The method of eyeball section was consistent with that in H&E. In briefly, the retinal slices were dewaxed, rehydrated, and then treated with the antigen repair kit, followed by washes with PBS. Endogenous peroxidase was blocked with hydrogen peroxide (H_2_O_2_, 3%) and incubated at 25° for 25 min before washed with PBS (PH7.4). Bovine serum albumin (BSA) was used for blocking (30 min at 25°C), primary antibody was added at 4°C overnight, and diluted secondary antibody (HRB marker) was added after the samples were washed with PBS. Then the samples were incubated at 30° for 1h. Images of retina were obtained by photographing near the optic disc (Eclipse C1, Nikon). One eyeball was taken from each of the six rats in each group for slice staining analysis.

### 2.13. Data analysis and visualization

The data were analyzed using student’s *t*-test. Differences with *p < 0.05 were considered statistically significant. Statistical analysis was performed as the mean ± standard deviation (SD). Data were analyzed and visualized by GraphPad Software or Origin 8.0 software.

## 3. Results

### 3.1. H_2_s relieves blue light-triggered retinal apoptosis and degeneration in vivo

**Figure 1.**
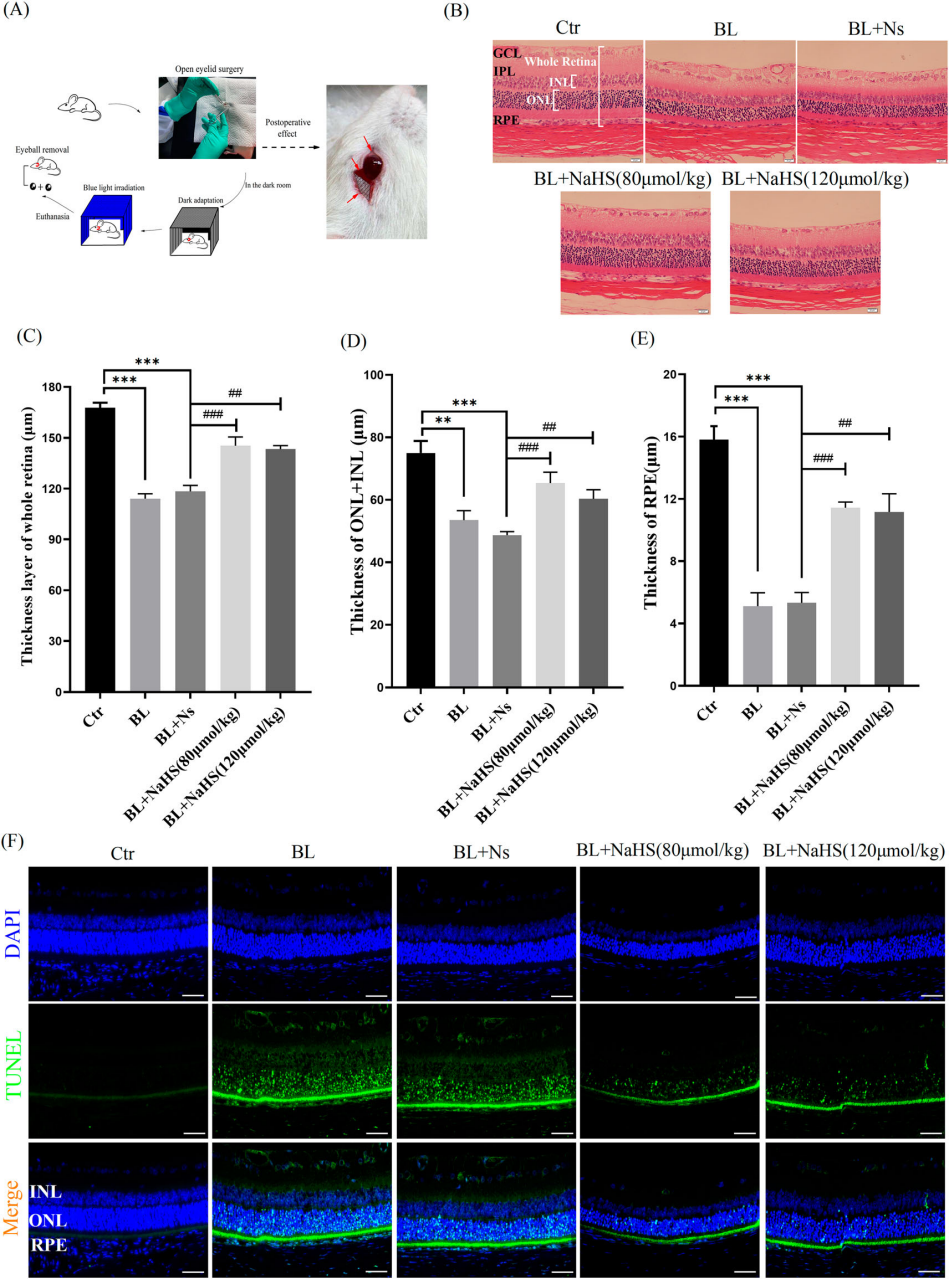
Exogenous H_2_S relieves blue light-induced retinal degeneration and apoptosis in rats. (A) Eyelid surgery procedure and its effects. (B) Retinal tissue morphology was detected by H&E staining (*n* = 6 eyes per group). (C) Statistics of whole retinal thickness. (D) Statistics of INL and ONL thickness. (E) Statistics of RPE cell layer thickness. (F) Retinal tissue sections of rats were stained with DAPI and TUNEL to detect apoptosis (*n* = 3 eyes per group). Ctr, control group. BL, blue light treatment. NS, normal saline. Values are mean ± SD. **p < 0. 01, ***p < 0.001 versus the control group; **^##^***p *< 0.01, **^###^***p *< 0.001 versus the BL + NS group.

The rats were undergone open-eyelid surgery to allow the retina to fully receive blue light (Figure 1(A) and Section 2.9). Retinal thickness is the guarantee of retinal function, and when the retina is damaged or degenerated, it will become thinner. We found that blue light significantly triggered retinal degeneration and thinning, including INL, ONL, and RPE cell layer (Figure 1(B–E)). Furthermore, exogenous H_2_S could restore the retinal thickness of rats which was damaged by blue light exposure (Figure 1(B–E)). This suggests that H_2_S can protect the retina from blue light-induced degeneration *in vivo*. Blue light-induced retinal cell apoptosis is an important factor in inducing retinal degeneration. Therefore, we further investigated whether H_2_S could resist blue light-triggered retinal degeneration through anti-apoptotic effect. The results showed that H_2_S indeed inhibited blue light-triggered retinal cell apoptosis *in vivo* (Figure 1(F)). All the evidence indicates that H_2_S can alleviate retinal degeneration through anti-apoptotic effect and thus play an important role in protecting the retina *in vivo*.

### 3.2. H_2_s inhibits blue light-triggered retinal oxidative stress and ER stress in vivo

**Figure 2.**
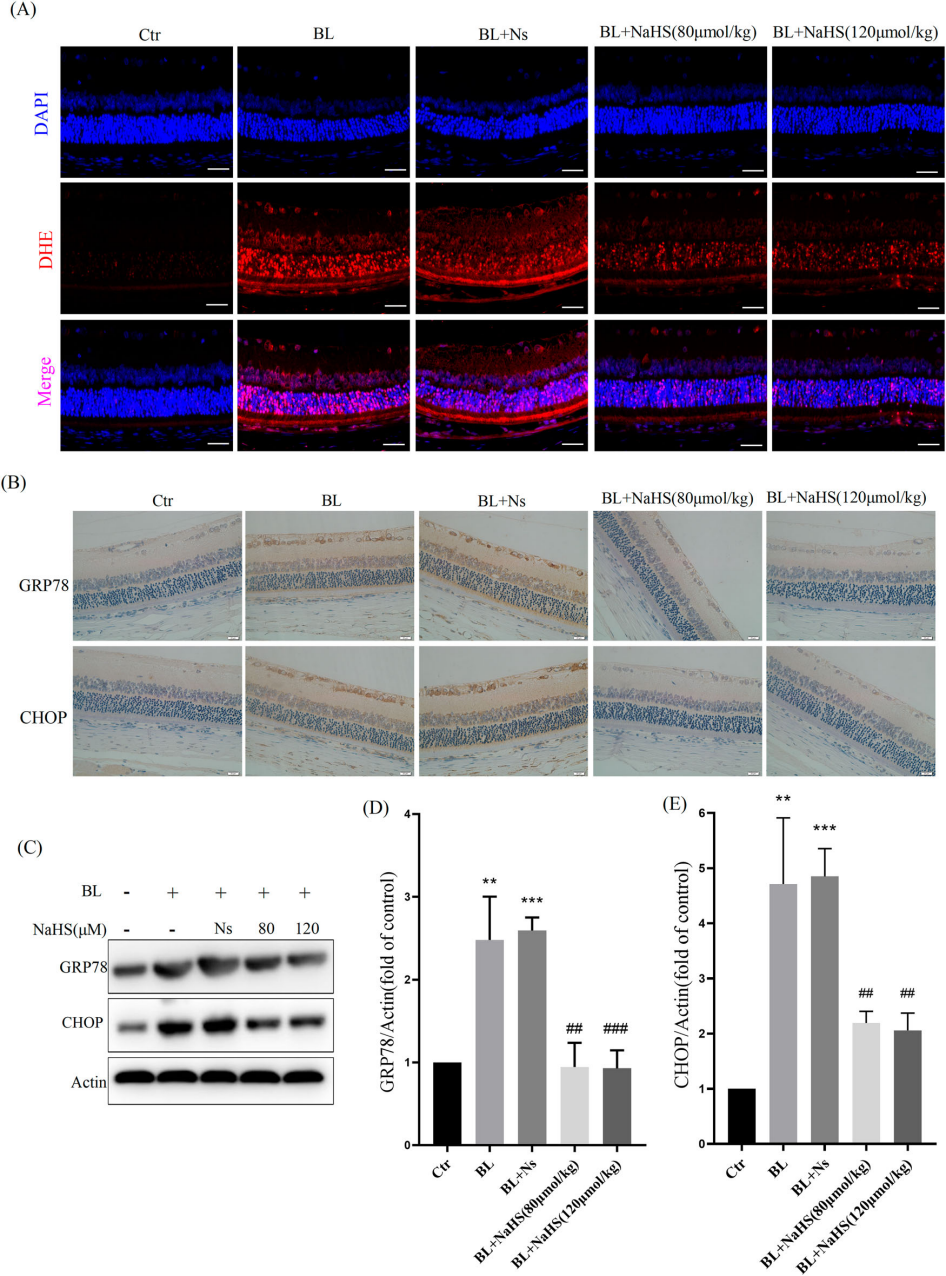
Exogenous H_2_S inhibits blue light-triggered retinal oxidative stress and ER stress *in vivo*. (A) ROS in the retina were labeled with the fluorescent probe DHE and detected by a fluorescent microscope (*n* = 3 eyes per group). (B) Expression of retinal CHOP protein was detected by immunohistochemistry (*n *= 6 eyes per group). (C) Western blot assay was used to detect the protein levels of GRP78 and CHOP in retinal tissue (*n* = 3 eyes per group). (D) Statistics of GRP78 protein alteration. (E) Statistics of CHOP protein alteration. Values are mean ± SD. **p < 0. 01, ***p < 0.001 versus the control group; **^##^***p *< 0.01, **^###^***p *< 0.001 versus the BL + NS group.

Studies have reported that ROS induced by blue light can further aggravate retinal apoptosis[9]. Therefore, we further studied the effect of H_2_S on blue light-induced retinal oxidative stress. DHE-labeled ROS in retinal cells, with DAPI-counterstained nucleus, were observed with a fluorescent microscope. The results showed that H_2_S could significantly inhibit the production of blue light-induced ROS (Figure 2(A)). In addition to inducing cellular oxidative stress, ROS can activate a variety of cell signals, including ER stress-CHOP apoptosis signal. We found that blue light induced the expression of CHOP and GRP78 in the retina of rats. Interestingly, H_2_S significantly inhibited blue light-induced activation of ER stress-CHOP apoptosis signal (Figure 2(B–E)). These results indicate that ER stress-CHOP apoptotic signal may be involved in blue light-triggered retinal apoptosis, which further induces retinal damage and degeneration *in vivo*. While exogenous H_2_S can inhibit oxidative stress and ER stress, thereby reducing blue light-triggered apoptosis and alleviating retinal degeneration.

### 3.3. H_2_s inhibits blue light-triggered oxidative damage in ARPE-19 cells

**Figure 3.**
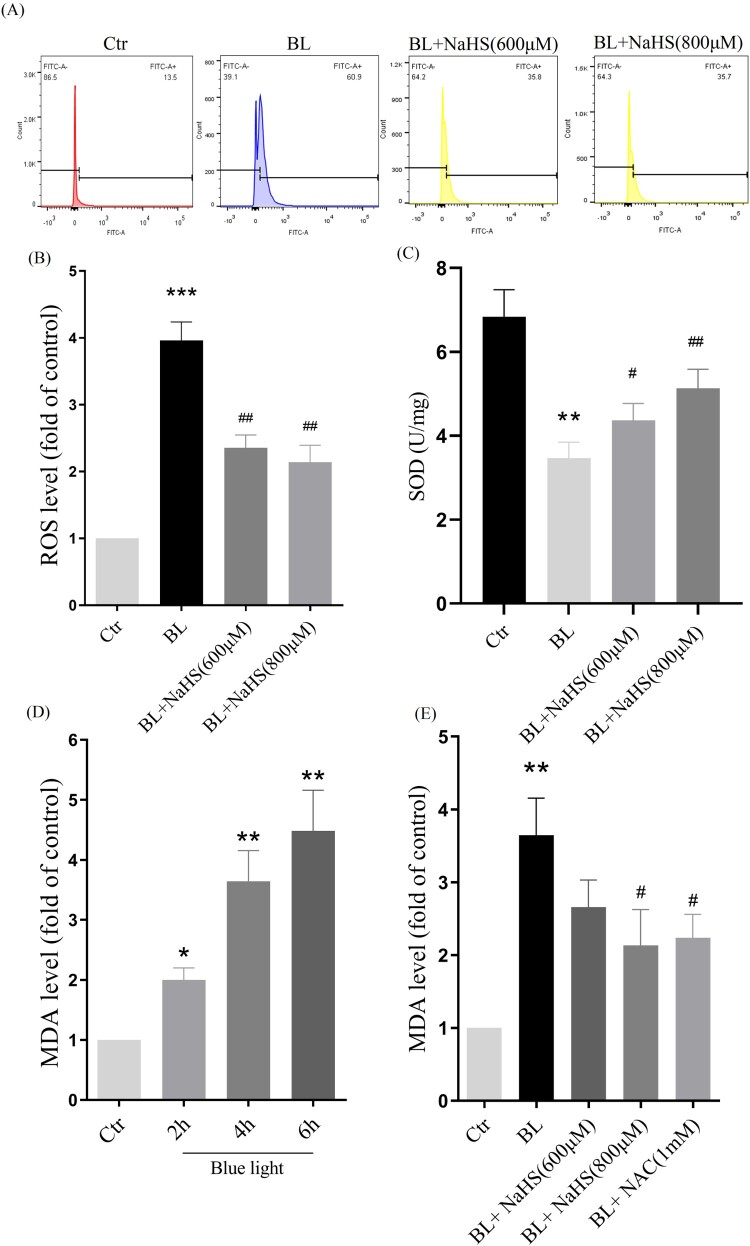
H_2_S inhibits blue light-triggered oxidative damage in ARPE-19 cells*.* (A) ARPE-19 Cells were pretreated with NaHS for 1h and then exposed to blue light for 4 h. Cells were stained with DCFH-DA, and intracellular ROS levels were observed by flow cytometry (*n* = 3). (B) Statistics of intracellular ROS level. (C) ARPE-19 Cells were pretreated with NaHS for 1 h and then exposed to blue light for 4 h, and then cultured for another 24 h. The intracellular SOD activity was detected by the assay kits (*n* = 3). (D) ARPE-19 cells were exposed to blue light for indicated time, and then cultured for another 24 h. The MDA assay kit used to detect MDA level in cells (*n* = 3). (E) ARPE-19 Cells were pretreated with NaHS for 1 h and then exposed to blue light for 4 h, and then cultured for another 24 h. The MDA assay kit was used to detect MDA level in cells (*n* = 3). Values are mean ± SD. *p < 0. 05, ** *p *< 0. 01, *** *p *< 0.001 versus the control group; **^#^** *p *< 0.05, **^##^** *p *< 0.01 versus the BL alone group.

To further confirm the effects of exogenous H_2_S, we have done a more detailed study *in vitro*. We found that H_2_S could significantly reduce blue light-induced ROS, and restore intracellular SOD activity (Figure 3(A–C)). In addition, we also detected the level of MDA, and the results showed that H_2_S could significantly alleviate blue light-triggered oxidative damage (Figure 3(D,E)). The above results indicate that H_2_S can resist blue light-triggered oxidative damage in ARPE-19 cells.

### 3.4. H_2_s inhibits blue light-induced ER stress-CHOP apoptotic signal activation in ARPE-19 cells

**Figure 4.**
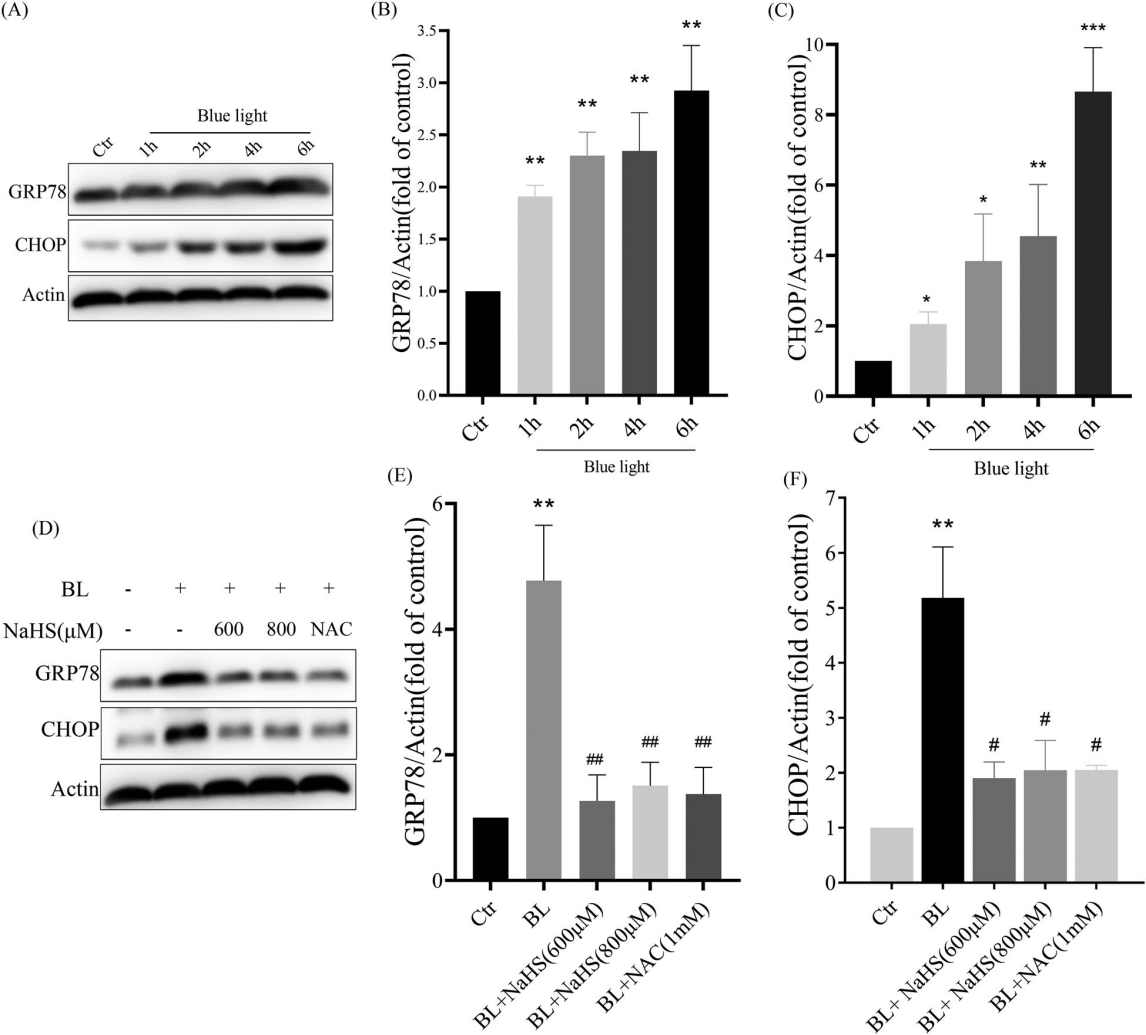
H_2_S inhibits blue light-induced activation of ER stress-CHOP apoptotic signal in ARPE-19 cells. (A) ARPE-19 cells were exposed to blue light for indicated time, and then cultured for another 24 h. Western blot assay was used to detect the protein levels of GRP78 and CHOP (*n* = 3). (B) Statistics of GRP78 protein alteration. (C) Statistics of CHOP protein alteration. (D) ARPE-19 Cells were pretreated with NaHS for 1 h and then exposed to blue light for 4 h, and then cultured for another 24 h. Western blot assay was used to detect the protein levels of GRP78 and CHOP (*n* = 3). (E) Statistics of GRP78 protein alteration. (F) Statistics of CHOP protein alteration. Values are mean ± SD. *p < 0. 05, **p < 0. 01, ***p < 0.001 versus the control group; **^#^***p *< 0.05, **^##^***p *< 0.01 versus the BL alone group.

Next, we studied the effect of H_2_S on blue light-induced ER stress-CHOP apoptosis signal. We found that blue light could induce the expression of CHOP and GRP78, and activate ER stress-CHOP apoptotic signal (Figure 4(A–C)), while exogenous H_2_S inhibited the expression of CHOP and GRP78 induced by blue light (Figure 4(D–F)). Similarly, we also found that another antioxidant NAC could inhibit blue light-induced activation of ER stress-CHOP apoptosis signal (Figure 4(D)). The evidence indicates that blue light-induced ROS activates the ER stress-CHOP apoptotic signal, while H_2_S inhibits the activation of ER stress-CHOP signal through its antioxidative effect.

### 3.5. ER stress-CHOP apoptosis pathway is involved in blue light-triggered apoptosis in ARPE-19 cells

**Figure 5.**
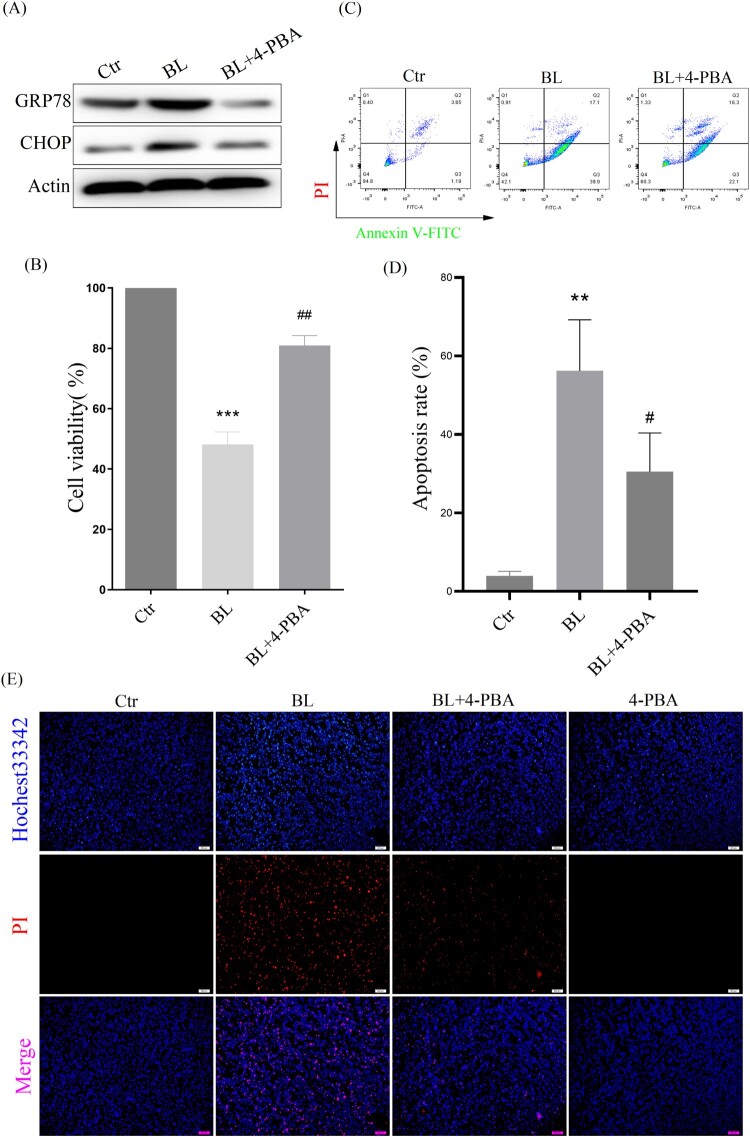
ER stress-CHOP apoptosis signal is involved in blue light-induced apoptosis in ARPE-19 cells. (A) ARPE-19 cells were pretreated with 4-PBA for 2 h and exposed to blue light for 4 h, and then cultured for another 24 h. Western blot assay was used to detect the protein levels of GRP78 and CHOP (*n* = 3). (B) MTT assay was used to analyze cell viability (*n* = 3). (C) Apoptosis was detected by flow cytometry with Annexin V-FITC and PI staining (*n* = 3). (D) Statistics of cell apoptosis. (E) Cell death was analyzed with Hochest33342 and PI staining, and observed under a fluorescent microscope (*n* = 3). Values are mean ± SD. **p < 0. 01, ***p < 0.001 versus the control group; **^##^** *p *< 0.01, **^###^***p *< 0.001 versus the BL alone group.

To verify whether ER stress-CHOP activation is involved in blue light-triggered apoptosis in ARPE-19 cells, the ER stress inhibitor 4-PBA was used. The results showed that 4-PBA suppressed the expression of GRP78 and CHOP induced by blue light (Figure 5(A)). When CHOP expression was inhibited, blue light-triggered apoptosis was significantly reduced, and cell survival was increased in ARPE-19 cells (Figure 5(B–D)). In addition, we also used Hochest33342/PI double staining to detect cell death, and found that when CHOP expression was inhibited, cell death was also significantly reduced (Figure 5(E)). All these results indicate that ER stress-CHOP apoptotic signal is indeed involved in blue light-triggered apoptosis in ARPE-19 cells.

### 3.6. H_2_s reduces blue light-triggered apoptosis in ARPE-19 cells

**Figure 6.**
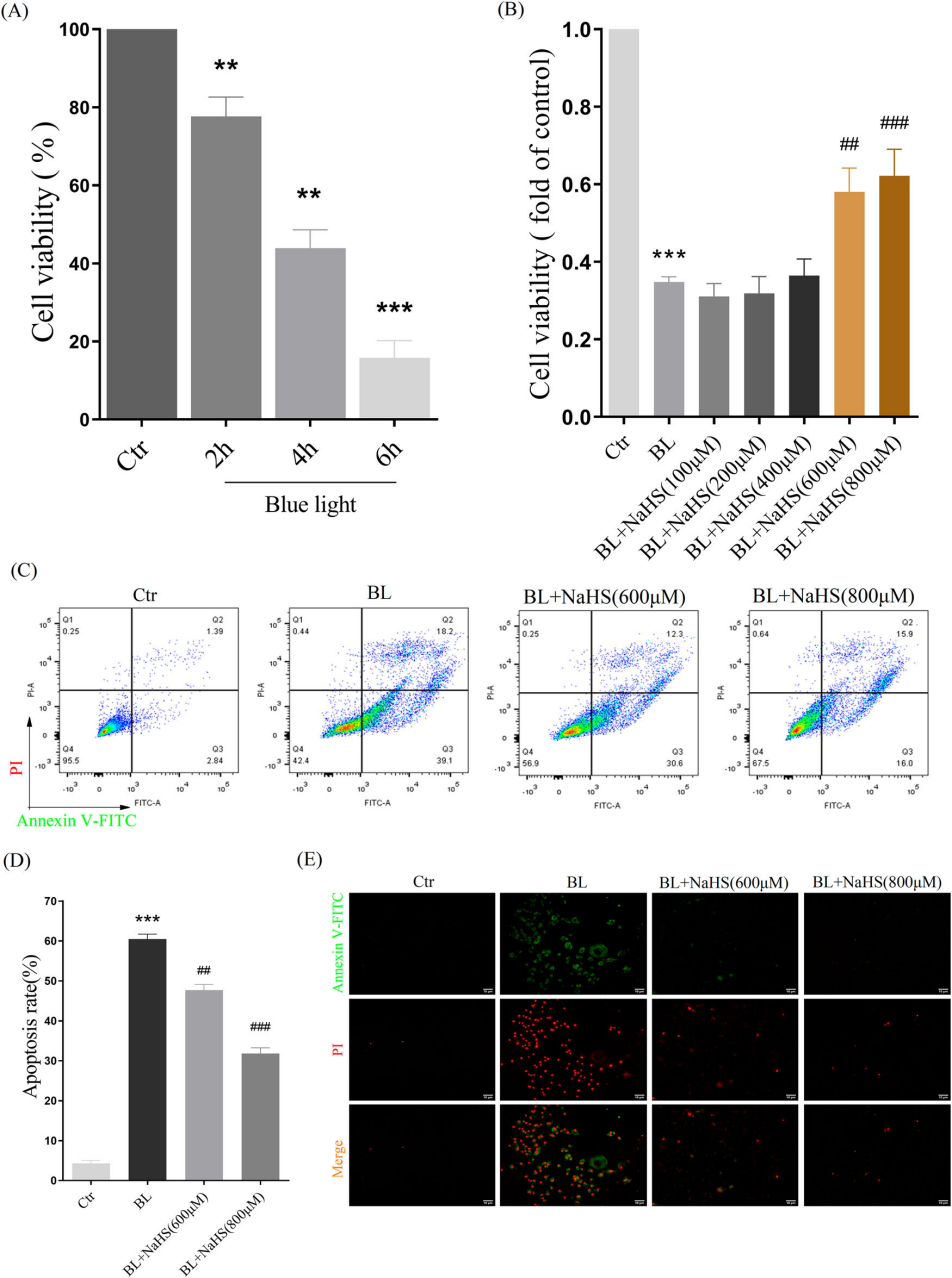
H_2_S reduces blue light-induced cell proliferation arrest and apoptosis in ARPE-19 cells. (A) ARPE-19 cells were exposed to blue light for indicated time, and then cultured for another 24 h. MTT assay was used to detect the cytotoxicity of blue light exposure (*n* = 3). (B) ARPE-19 cells were pretreated with NaHS for 1 h and exposed to blue light for 4 h, and then cultured for another 24 h. MTT assay was used to detect cell viability (*n *= 3). (C) Apoptosis was detected by flow cytometry with Annexin V-FITC and PI staining (*n* = 3). (D) Statistics of cell apoptosis. (E) Apoptosis was detected by a fluorescent microscope with Annexin V-FITC and PI staining (*n* = 3). Values are mean ± SD. **p < 0. 01, *** *p *< 0.001 versus the control group; **^##^** *p *< 0.01, **^###^** *p *< 0.001 versus the BL alone group.

Then, we further studied the effect of H_2_S on blue light-induced proliferation arrest and apoptosis in ARPE-19 cells. The results showed that blue light induced proliferation arrest in ARPE-19 cells, in a time-dependent manner (Figure 6(A)). While H_2_S pretreatment significantly alleviated this proliferation arrest (Figure 6(B)). Furthermore, we also found that H_2_S indeed inhibited blue light-induced apoptosis in ARPE-19 cells (Figure 6(C,D)). Consistent with this, the results of apoptosis, observed under a fluorescent microscope, also confirmed the protective effect of H_2_S on retinal ARPE-19 cells *in vitro* (Figure 6(E)).

## 4. Discussion

With the increasing use of modern electronic devices, more and more teenagers are experiencing vision loss [15]. Most previous studies have suggested that vision loss is caused by myopia. People have obviously ignored the effects of blue light emitted by electronic products [16]. Blue light is the second most powerful form of sun light after ultraviolet, and its penetration ability is stronger than that of ultraviolet, and blue light at wavelengths of 415–445 nm is the most harmful [17]. The retina is the tissue that consumes oxygen very fast, and external stimuli can easily lead to oxidative stress in the retina [18]. Blue light can induce the increase of ROS in ARPE-19 cells and cause cell oxidative stress and oxidative damage (Figure 3). Blue light can also inhibit cell proliferation and induce apoptosis in ARPE-19 cells (Figures 5–6). Our *in vivo* evidence has also shown that blue light triggers retinal oxidative stress (Figure 2), and induces retinal degeneration and apoptosis in rats (Figure 1). Previous studies also show that blue light can induce retinal degeneration, which is mainly characterized by reduced retinal thickness and structural disorders *in vivo* [19]. It is indicated that blue light-induced retinal apoptosis is a crucial impact factor to retinal photodamage and degeneration [20].

However, previous studies cannot eliminate experimental errors that the retina in each animal is possible to receive different blue light radiation dose, due to that different animal (including rats and mice) may close their eyes differently when they are exposed to blue light. To exclude this possibility, the rats were undergone open-eyelid surgery in this study, to allow the retina to fully receive blue light (Figure 1A and Section 2.9). The operation did not damage the ocular muscle tissue, lacrimal sac and lacrimal gland in rats, and according to our observations, the blink reflex was not affected after the recovery period. This operation can maximize the exposure of the eyeball while maintaining the normal protective function of the eye, so that the retina can receive sufficient blue light radiation dose.

Hydrogen sulfide is a small reducing molecule, which plays an important role in the treatment and prevention of a variety of physiological and pathological diseases [21,22], including retinal related diseases [23]. H_2_S can play a protective role by anti-oxidative, anti-inflammatory and anti-apoptotic effects [24]. In our previous research, we have found that H_2_S can significantly relieve the symptoms of age-dependent macular deformation (AMD), which is closely related to the anti-oxidative, anti-inflammatory and anti-apoptotic effects of H_2_S [14]. Therefore, we have further studied the effect of H_2_S on blue light-induced retinal damage and degeneration. Exogenous H_2_S significantly inhibits the production of ROS induced by blue light, reduces the level of MDA and improves the activity of the antioxidant enzyme SOD in ARPE-19 cells (Figure 3). H_2_S can also relieve blue light-induced cell proliferation arrest, and reduce cell apoptosis and death (Figure 6). Furthermore, the rats were intraperitoneally injected with H_2_S (NaHS as a donor) and then exposed to blue light for 8 h. Exogenous H_2_S significantly reduces blue light-induced retinal oxidative stress in rats (Figure 2(A)), hence reducing retinal apoptosis and restoring retinal thickness (Figure 1), indicating that exogenous H_2_S protects the retina from blue light-triggered damage and degeneration. Therefore, both *in vivo* and *in vitro* results confirm that H_2_S has a protective effect on blue light-induced retinal damage and degeneration.

Next, we have further studied the mechanism by which exogenous H_2_S protects the retina from blue light-induced apoptosis and degeneration. The ER is the main place for protein folding and processing. When the ER is disturbed by external factors, misfolded and unfolded proteins will accumulate in ER, which activates the ER stress response to assist proteins to fold and assemble correctly [25]. However, the continuous cellular stress response can induce the expression of CHOP protein and thus induce cell apoptosis [26]. Previous studies have shown that oxidative stress-mediated ER stress-CHOP apoptosis signal may be involved in blue light-induced apoptosis in ARPE-19 cells [27]. Interestingly, we have found that exogenous H_2_S significantly inhibits the expression of CHOP in ARPE-19 cells (Figure 4) and in rat retina (Figure 2). Similarly, another antioxidant NAC suppresses the expression of GRP78 and CHOP induced by blue light (Figure 4(D–F)), and the ER stress inhibitor 4-PBA also relieves blue light-induced cell proliferation arrest and reduces cell apoptosis and death (Figure 5). These data confirm that exogenous H_2_S protects the retina from blue light-induced damage and degeneration, by inhibiting blue light-induced ROS-mediated ER stress-CHOP apoptosis signal.

In the detection of ROS in retina, due to the specificity of ROS, we have to use the cryosection method. The eyeballs were cut into 5 μm slices along the vertical meridian of eyeballs. Images of retinal ROS were obtained by photographing near the optic disc. However, we should admit that compared with paraffin sections, frozen sections do not have advantages in preserving original tissue morphology and structure, which may be the reason for the differences in bending degree between different tissues.

In summary, this study demonstrates the protective effect of H_2_S on blue light-induced retinal damage and degeneration *in vivo* and in *vitro*, and reveals its protective mechanism by inhibiting ROS-mediated ER stress-CHOP apoptosis signal, indicating that exogenous H_2_S has a great potential in protecting the retina from oxidative damage and its related diseases. However, we must emphasize that since these data are obtained from cell and rat experiments, whether they can be used in the clinic requires more experiments and evidence in the future.

## Ethics approval and consent to participate

This study on animals was conducted according to the guidelines of the Declaration of Helsinki, and approved by the Ethics Committee of School of Life Sciences, Lanzhou University (EAF2021001).

## Submission declaration

We declare that its publication has been approved by all the authors.

## Supplementary Material

Supplemental Material

## Data Availability

The data used to support the findings of this study are available from the corresponding author upon request.
