# Supplementary material for: Hydrogen sulfide protects retina from blue light-induced photodamage and degeneration via inhibiting ROS-mediated ER stress-CHOP apoptosis signal
Source: Redox Rep. 2022 Apr 28;27(1):100–10. doi: 10.1080/13510002.2022.2069534 (PMC9067965; doi:10.1080/13510002.2022.2069534)
Supplement: Supplemental Material [file YRER_A_2069534_SM4358.docx]

**Material and Methods**

*1. The effect of NaHS on the pH value of the medium.* NaHS was dissolved in DMEM medium to make 100× stock solution, diluted and added to complete medium (90%DMEM+10%FBS), and then the change of medium pH value was detected at different times (0,5,10,30,60 min).

**FIGURE S1**

*
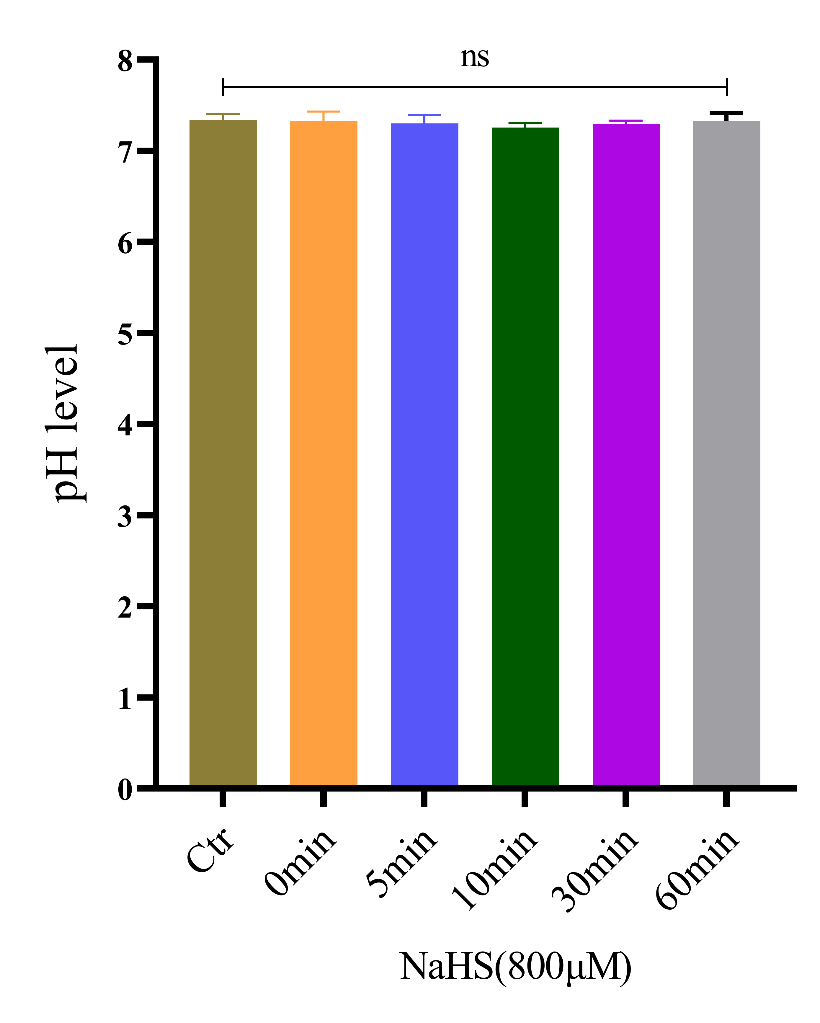
*

**Figure legends**

**Figure S1:** The changes of pH value after adding NaHS into complete medium. Exogenous H_2_S (NaHS as donor) did not have obvious effect on the pH value of cell culture medium.
